# Megf10‐related engulfment of excitatory postsynapses by astrocytes following severe brain injury

**DOI:** 10.1111/cns.14223

**Published:** 2023-04-20

**Authors:** Yuan Zhuang, Xiaojian Xu, Hao Li, Fei Niu, Mengshi Yang, Qianqian Ge, Shenghua Lu, Yu Deng, Hongbin Wu, Bin Zhang, Baiyun Liu

**Affiliations:** ^1^ Department of Neurosurgery, Beijing Tiantan Hospital Capital Medical University Beijing China; ^2^ Beijing Key Laboratory of Central Nervous System Injury Beijing Neurosurgical Institute, Capital Medical University Beijing China; ^3^ Department of Intensive Care Unit, Beijing Tiantan Hospital Capital Medical University Beijing China; ^4^ Center for Nerve Injury and Repair Beijing Institute of Brain Disorders Beijing China; ^5^ China National Clinical Research Center for Neurological Diseases Beijing China

**Keywords:** astrocytes, hippocampus, Megf10, phagocytosis, traumatic brain injury

## Abstract

**Aims:**

To investigate astrocyte‐related phagocytosis of synapses in the ipsilateral hippocampus after traumatic brain injury (TBI).

**Methods:**

We performed controlled cortical impact to simulate TBI in mice. Seven days postinjury, we performed cognitive tests, synapse quantification, and examination of astrocytic phagocytosis in association with Megf10 expression.

**Results:**

During the subacute stage post‐TBI, we found a reduction in excitatory postsynaptic materials in the ipsilateral hippocampus, which was consistent with poor performance in the cognitive test. The transcriptome data suggested that robust phagocytosis was responsible for this process. Coincidently, we identified phagocytic astrocytes containing secondary lysosomes that were wrapped around the synapses in the ipsilateral hippocampus. Moreover, a significant increase in the co‐location of GFAP and PSD‐95 in the CA1 region suggested astrocytic engulfment of excitatory postsynaptic proteins. After examining the reported phagocytic pathways, we found that both the transcription level and protein expression of Megf10 were elevated. Co‐immunofluorescence of GFAP and Megf10 demonstrated that the expression of Megf10 was spatially upregulated in astrocytes, exclusively in the CA1 region, and was related to the astrocytic engulfment of PSD‐95.

**Conclusion:**

Our study elaborated that the Megf10‐related astrocytic engulfment of PSD‐95 in the CA1 region of the ipsilateral hippocampus aggravated cognitive dysfunction following severe TBI.

## Graphical abstract

7 days after TBI, Megf10‐related astrocytic phagocytosis of postsynaptic materials was significantly upregulated in the CA1 of the ipsilateral hippocampus.

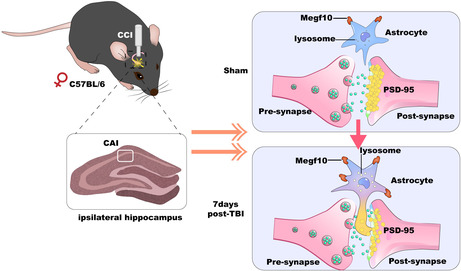

## 1 INTRODUCTION

Severe traumatic brain injury (TBI) is a major health concern. In addition to the primary attack, continuous secondary injuries have widespread implications for the entire brain circuitry. The complex mechanisms of these secondary injuries result in neurological deficits, behavior alternation, and continuous cognitive impairment.^1^ Understanding the TBI‐related pathological process is crucial for improving neurotherapeutics. Among the various brain regions, the hippocampus plays a vital role in cognition and is vulnerable to TBI. Clinical studies have shown that hippocampus volume is correlated to TBI severity and neurological deficits.^2^, ^3^ Following TBI, both apoptosis and inflammation occur, as well as the reorganization of synaptic neural circuits in the hippocampus, which may be a promising target for interventions to improve TBI‐related cognitive impairment.

Astrocytes are composed of typical glial cells and respond to various biological processes. Because they wrap around synapses, astrocytes are an important contributor to synaptic transmission. Recent studies have proposed that astrocytes contribute to synapse formation and the development of the central nervous system (CNS) by secreting TSP, Hevin, GPC4, and GPC6.^4^, ^5^, ^6^ Furthermore, transcriptomic profiling has shown that astrocytes are enriched with synaptic engulfment pathway‐related genes, which determine their phagocytic properties.^7^ In the adult hippocampus, astrocytic phagocytosis results in synaptic elimination and helps to maintain synaptic connectivity and plasticity.^8^ Although previous studies have demonstrated astrocytic engulfment in apoptotic debris, whether it causes synaptic loss following TBI remains unclear.^7^

Multiple‐EGF‐like domains 10 (Megf10) is a type I transmembrane protein that is expressed in macrophages and glial cells. Previous studies have indicated that Megf10 is abundantly expressed in astrocytes, mediates synaptic elimination and cell type‐specific recognition, and cooperates with adenosine triphosphate binding cassette transporter I (ABCA1).^9^ A study by Chung et al.^10^ revealed that astrocytes engulf synapses via the Megf10 and Mertk pathways in both developmental and adult brains. Lee et al.^8^ also confirmed that astrocytic Megf10 mediates the elimination of excitatory synapses in the CA1 region of the adult hippocampus in an activity‐dependent manner, which is important for maintaining hippocampal synaptic connectivity. Conversely, poststroke reactive astrogliosis associated with Megf10‐mediated engulfment of synapses aggravates neurological impairment.^7^

Traumatic brain injury‐related pathology is complex and is accompanied by robust astrogliosis and synaptic plasticity. To date, the influence of astrocytes on synapses post‐TBI has not yet been clarified. Therefore, in the present study, we examined Megf10‐related robust engulfing of synapses by astrocytes in the ipsilateral hippocampus following severe TBI and how it contributes to postinjury cognitive deficits. We hypothesized that intervening in the engulfment of astrocytes would be a promising target for treatment following TBI.

## 2 MATERIALS AND METHODS

### 2.1 Animals

Specific pathogen‐free (SPF) male adult C57BL/6 mice (weighing 20–25 g, *n* = 60) were obtained from the Beijing Vital River Laboratory Animal Technology Co., Ltd. All the experiments were approved by the local animal ethics committee (approval no. 202103002). The experiments were designed and reported according to the Animal Research: Reporting of In Vivo Experiments guidelines. All the mice were housed in a controlled environment (five animals per cage; 12‐h light/dark cycle; temperature, 22 ± 2°C; humidity, 50%–60%) with food and water ad libitum. Mice were acclimated for 1 week before being randomly divided into the sham or TBI group (7 days after the controlled cortical impact [CCI]).

### 2.2 CCI model

The CCI model was applied to the mice in the TBI group to induce TBI, the details of which have been reported in our previous studies.^11^, ^12^ Mice were anesthetized with 5% isoflurane (RWD Life Science Co., Ltd.) in 100% oxygen at a delivery rate of 1 L/min, and maintenance anesthesia was then administered using 2% isoflurane inhalation (Sigma). The animals were fixed in a prone position, and the skull was exposed using a midline incision. We then performed a 4.0‐mm diameter craniotomy over the right parietal lobe using an electric drill (RWD Life Science Co.), leaving the underlying dura intact. Subsequently, mice were subjected to CCI with a 3.0‐mm diameter flat tip fixed onto an electromagnetic CCI device (Pinpoint PCI3000 Precision Cortical Impactor, Hatteras Instruments) at a velocity of 3.0 m/s, a depth of 1.5 mm, and a 20‐ms dwelling time. The CCI model was considered successful when a noticeable cerebral cortical contusion was observed. After careful hemostasis, the trauma site was sealed using bone wax, and the skin was sutured using 4‐0 nylon wire.

### 2.3 Behavioral tests

#### 2.3.1 Elevated plus maze (EPM) test

Seven days after CCI, both groups underwent the EPM test to evaluate cognitive and memory abilities.^13^, ^14^, ^15^ The EPM apparatus contained two open (OAs; 100 × 10 cm) and two closed arms (CAs; 100 × 10 cm with 40‐cm high walls) that were placed opposite to each other and a central platform (10 × 10 cm). The maze was placed 50 cm above the floor. Animals that fell off the maze or failed to enter the CA within 60 s were excluded from the experiment. During the test, animals were placed in the center platform where the arms intersect and were allowed to explore freely for 5 min. All the procedures were recorded using an AHD camera. The maze was cleaned with 75% ethanol between trials to eliminate clues. The speed ratio of the CA to OA, residence time, and distance traveled in the OA were analyzed to compare cognition between the two groups.^16^

#### 2.3.2 Novel object recognition test (NORT)

Mice exhibit habitual behavior of exploring a new object. Therefore, we used the NORT to compare cognitive changes between the two groups. The NORT contains three phases: habituation, familiarization, and discrimination. The habituation lasted 3 days, whereby each mouse was placed in a square open field (50 × 25 × 10 cm) for 5 min. During the familiarization stage, each mouse was allowed to explore the open field, which contained two identical triangular objects (A1 and A2) located in opposite and equidistant positions, for 5 min. Finally, during the discrimination stage, mice were returned to the open field, which contained one familiar object (A2) and a novel round object (A3), and mice were allowed to explore the field for 5 min. Touching or remaining 2 cm from the novel object was considered object exploration behavior. The object and field were cleaned with 75% ethanol between trials. The discrimination index (DI) was calculated by (A3 − A1)/*t*(A3 + A1).^17^

### 2.4 Quantitative reverse transcription polymerase chain reaction (qRT–PCR)

Total RNA was extracted from the ipsilateral hippocampus using a Trizol reagent (Biosharp, cat. BS259A) according to manufacturer instructions. The RNA was then reverse‐transcribed to cDNA using SuperScript II Reverse Transcriptase. (Thermo Fisher, cat 4368814) We then conducted qRT–PCR in a 20‐μL reaction volume using SYBR Premix Ex Taq (Thermo Fisher, cat 4367659). The threshold cycle readings were collected, and the relative expression of the target gene mRNA was calculated using the 2^−ΔΔCT^ method. Relative expression was normalized to the β‐actin level. The target mRNA expression level was analyzed as a fold change and was compared between the sham and TBI groups. The sequences of primer pairs for the target genes were as follows:

GFAP: forward 5′‐ AGATTCGCACTCAATACGAGG‐3′

Reverse 5′‐ CTGTGAGGTCTGCAAACTTAGA‐3′

Dlg4: forward 5′‐ TCCAGTCTGTGCGAGAGGTAGC‐3′

Reverse 5′‐ GGACGGATGAAGATGGCGATGG‐3′

Slc17A7: forward 5′‐ TTGTGGCTACCTCCACCCTAA‐3′

Reverse 5′‐ CAGCCGACTCCGTTCTAAGG‐3′

Megf10: forward 5′‐ GAAGACCCCAACGTATGCAG‐3′

Reverse 5′‐ CGGTGCAGCTTGTGTAGTAGA‐3′

Mertk: forward 5′‐ GAAGGAGAGTTTGGGTCTGTAA‐3′

Reverse 5′‐ GTTGTCCAACTTCATGGTCTTC‐3′

β‐actin: forward 5′‐ GTGCTATGTTGCTCTAGACTTCG‐3′

Reverse 5′‐ ATGCCACAGGATTCCATACC‐3′

### 2.5 Western blot (WB) analysis

The ipsilateral hippocampus tissue was collected for WB analysis using gradient centrifugation, as described previously.^18^ Protein concentration was assessed using the BCA method, and 30 μg of extract was separated using sodium dodecyl‐sulfate polyacrylamide gel electrophoresis (SDS–PAGE). The separated proteins were then transferred onto polyvinylidene fluoride membranes (PVDFs). The PVDF membranes were incubated with 5% skim milk for 1 h at room temperature and subsequently allowed to react overnight at 4°C with the following primary antibodies: GFAP (1:1000), PSD‐95 (1:1000), vGlut1 (1:1000), Megf10 (1:1000), and Abca1 (1:1000). The PVDF membranes were then incubated with corresponding secondary antibodies (1:5000) for 1 h at the room temperature. The blot was visualized with chemiluminescence (Amersham Imager 600, GE). The relative band density was measured using the ImageJ software (version 1.53a) and normalized to the band density of GADPH.

### 2.6 Golgi staining

The Golgi‐staining process used is reported in a previous study.^19^ Mice were decapitated, and brain tissue was removed 7 days after CCI. Golgi staining of brain tissue was performed using the FD Rapid Golgi Stain™ Kit (FD Neuro Technologies, Inc.) according to manufacturer instructions. Dendrites in the ipsilateral hippocampus were identified using a 63× magnification lens, and dendritic density was calculated using the ImageJ software (version 1.53a).

### 2.7 Electron microscopy (EM)

Mice were perfused with saline before ipsilateral hippocampus specimens were collected. Brain tissue was fixed in 2% glutaraldehyde in 0.1 M phosphate buffer and reacted with 1% osmium tetroxide in 0.1 M phosphate buffer. Slices were then dehydrated in a graded ethanol series, infiltrated with propylene oxide, and embedded in Spurr's epoxy resin. Before observation, thin sections were cut using an ultramicrotome and stained with uranyl acetate and lead citrate. We then observed the morphology of astrocytes and the positional relationship between astrocytes and synapses.

### 2.8 Immunofluorescence staining

The procedure for immunofluorescence staining is described in previous studies.^19^ The following primary antibodies were used: GFAP (1:500, rabbit, cat # ab279290, Abcam); PSD‐95 (1:500; mouse, MA1‐046, Thermo); Megf10 (1:200; rabbit, 32160702, Sigma); LAMP2 (1:200; rabbit, YT5711, Imunoway). Tissues were incubated at 4°C for 2 days, followed by incubation with the corresponding secondary antibodies of Alexafluor 488 or 594 (1:500, Abcam) for 1 hour at room temperature. Samples were mounted using Vectashield containing DAPI for cell nuclei staining (ab104139, Abcam).

### 2.9 Hematoxylin–eosin (HE) staining

Brain tissues were fixed with 4% paraformaldehyde, paraffin‐embedded, and cut into 3‐μm coronal sections. HE staining was carried out according to standard protocols, which comprised dewaxing, rehydrating, hematoxylin staining, eosin staining, dehydrating, and hyalinizing.^20^

### 2.10 Differentially expressed gene (DEG) analysis

Total RNA extraction, mRNA library construction, sequencing, and acquisition of clean data were performed by Genergy Biotechnology Co. Ltd. DEG analysis was performed using the DESeq2 software. Paired genes were considered differentially expressed using the following standards: false discovery rate (FDR) < 0.05 and absolute fold change ≥ 2.

### 2.11 Gene ontology (GO) and Kyoto Encyclopedia of Genes and Genomes (KEGG) pathway enrichment analyses

The functions of DEGs were depicted using GO (http://geneontology.org) and the KEGG pathway analyses (http://www.genome.jp/kegg). A *p* < 0.05 indicated significant enrichment.

### 2.12 Gene set enrichment analysis (GSEA)

Gene set enrichment analysis was used to identify significantly different genes in specific KEGG pathways.

Mice genes were assigned to corresponding human orthologs using the GSEA “collapse dataset to gene symbols” feature using the respective symbol remapping chip (Mouse_Gene_Symbol_Remapping_Human_Orthologs_MSigDB.v7.5.1.chip), and the GSEA software (version 4.2.3) was used to determine canonical pathway enrichment (i.e., the C2 KEGG subset of canonical pathways). |NES| > 1, NOM *p*‐value <0.05, and FDR *q*‐value <0.25 were considered the selected conditions.

### 2.13 Gene set variation analysis (GSVA)

Gene set variation analysis was used to estimate the altered pathways vulnerable to TBI. Because this method is nonparametric and nonsupervised, it provides additional details with subtle alternation of pathway changes. The R packages of GSVA (1.45.2) and Limma (3.53.3) were used to determine the differentially expressed KEGG pathways.

### 2.14 Statistical analysis

All the data are presented as means ± standard deviations. The normality of the data was assessed using the Shapiro–Wilk test and *t*‐tests were used to compare the two groups. Significance was defined as *p* < 0.05. All statistical analyses were performed using SPSS version 23.0 (IBM), and graphs were generated using the GraphPad Prism 9 software.

## 3 RESULTS

### 3.1 Cognitive dysfunction caused by unilateral severe TBI

**FIGURE 1:**
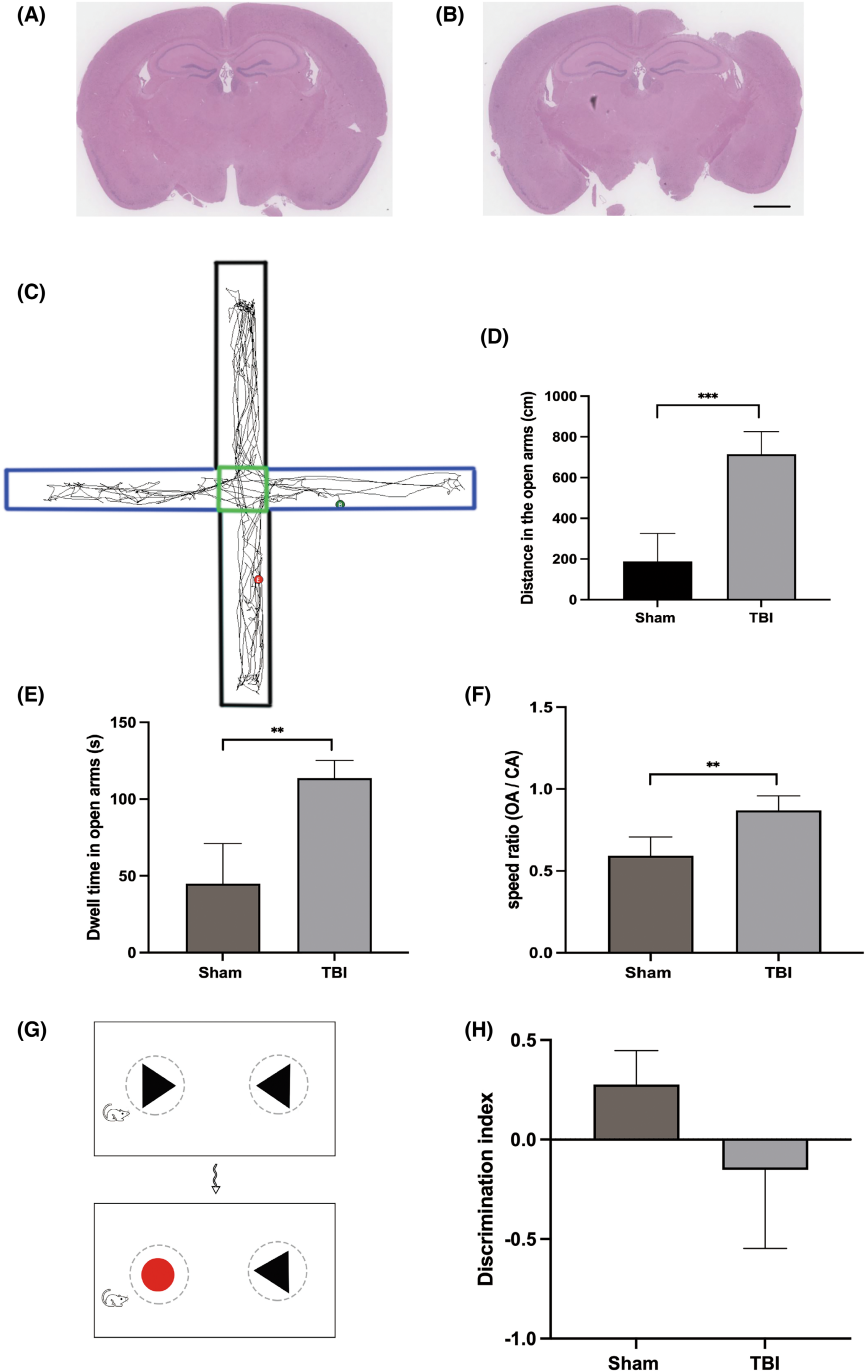
TBI was induced using the right CCI model, which resulted in cognitive impairment. (A and B) HE staining of hippocampal tissue in the sham (A) and TBI (B) groups. We observed deep lesions above the ipsilateral hippocampus, but the hippocampus remained intact. Scale bar: 4 mm. (C) The EPM test comprised a square with two OAs (blue) and two CAs (black). (D) Distance traveled in the OAs by mice in the TBI group was longer than that in the sham group (714.38 ± 111.24 cm vs. 188.56 ± 136.89 cm; ***p = 0.001). (E) Mice in the TBI group showed a longer dwell time in the OAs than in the sham group (113.61 ± 11.55 s vs. 44.84 ± 26.24 s; **p = 0.003). (F) The OA/CA speed ratio of mice in the TBI group was larger than that in the sham group (0.87 ± 0.89 vs. 0.59 ± 0.11; **p = 0.003). (G) Schematic of the NORT. After familiarizing the mice with the two triangle objects, a circular object replaced one of the triangle objects. (H) The discrimination index, a measure of recognition ability, of mice after TBI was lower than that of mice in the sham group (−0.15 ± 0.40 vs. 0.28 ± 0.17; *p = 0.035).

Traumatic brain injury in the right parietal lobe (Figure 1A,B) resulted in secondary sterile inflammation in the hippocampus, which critically affected cognitive functions. To assess the cognitive changes, we performed the EPM test and the NORT. Mice in the sham group showed anxiety in the OAs, as indicated by shorter residence times and travel distances, as well as lower speed ratios. However, anxiety was not seen after TBI, indicating cognitive dysfunction. (Figure 1C,F).

Our results showed that TBI‐impaired recognition memory. Mice in the sham group preferred the novel object more than those in the TBI group. Compared with the sham group, TBI mice showed poorer recognition of the novel object and tended to stay near the familiar object 7 days after TBI. (Figure 1G,H).

### 3.2 Robust engulfment in the ipsilateral hippocampus 3 days after TBI

**FIGURE 2:**
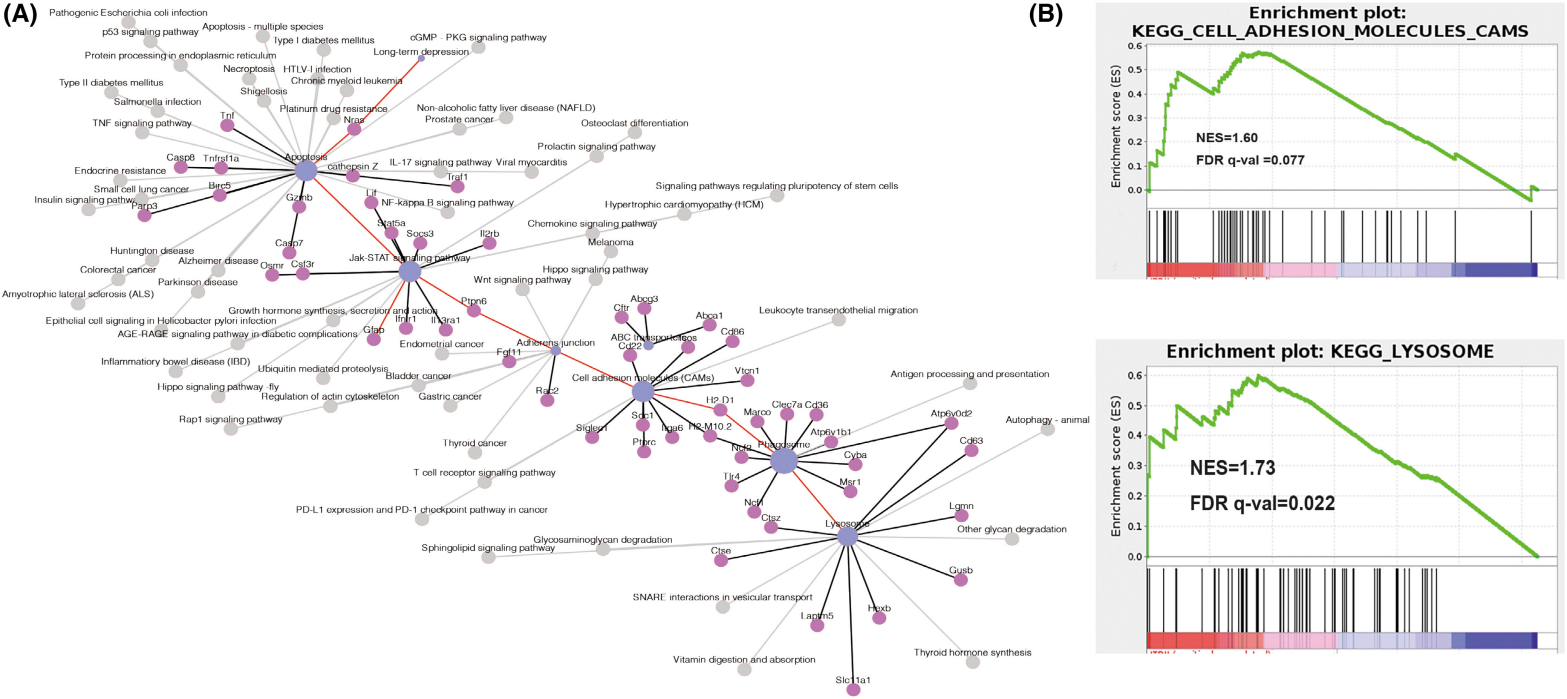
Transcriptome analysis of the ipsilateral hippocampus 3 days post‐TBI showed hyperactive phagocytosis. (A) The KEGG network showed that TBI‐induced phagocytosis was related to lysosomes, cell adhesion, and apoptosis. (B) GSEA analysis demonstrated that the elevated genes were enriched in phagocytotic processes related to lysosomes and cell adhesion.

We previously conducted transcriptome analysis in the ipsilateral hippocampus 3 days after TBI. We found that 1036 genes were upregulated and related to inflammation and cell growth (Figure S1A). Functional enrichment as assessed by the GO and KEGG analyses showed that DEGs were enriched in the synaptic process and the apoptotic, protein digestion, and absorption pathways (Figure S1B,C). Further investigation of the KEGG network indicated that molecular adhesion and phagocytosis played vital roles throughout the hippocampus pathological process following TBI (Figure 2A). We then used two unsupervised approaches of GSEA and GSVA to detect the subtle reactivated pathways. As expected, cell adhesion and lysosome pathways were significantly upregulated (Figure 2B; Figure S1D). These transcriptome results suggested that robust phagocytosis in the ipsilateral hippocampus is responsible for the cognitive changes following TBI and may be a promising target for intervention.

### 3.3 TBI resulted in a synaptic reduction in the ipsilateral hippocampus

**FIGURE 3:**
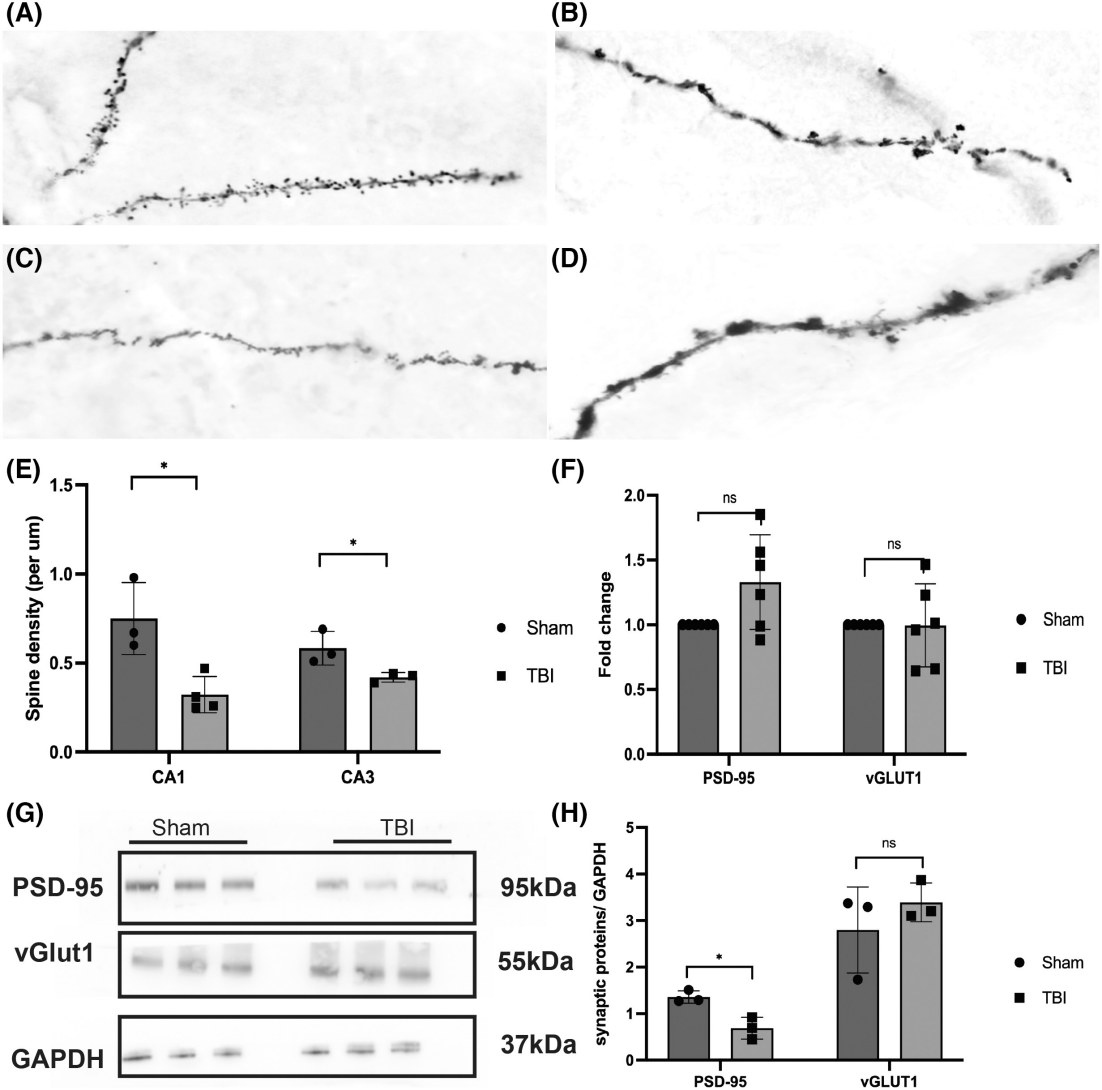
TBI resulted in a reduction in spines and excitatory post‐synaptic materials in the ipsilateral hippocampus. (A and B) Spines in the CA1 region of the ipsilateral hippocampus in the sham and TBI groups, respectively. (C and D) Spines in the CA3 of the ipsilateral hippocampus in the sham and experimental groups, respectively. (E) Spine density significantly reduced in both the CA1 and CA3 regions of the ipsilateral hippocampus 7 days post‐TBI (0.32 ± 0.10 vs. 0.75 ± 0.20, *p = 0.14 in CA1; 0.42 ± 0.24 vs. 0.58 ± 0.95, *p = 0.048 in CA3). (F) qRT‐PCR showed no difference in the transcript level of PSD‐95 (Dlg4) or vGlut1(Slc17a7) between the TBI and sham groups (Dlg4: 1.33 ± 0.37 vs. 1.00 ± 0.00, *p* = 0.051; vGlut1: 1.00 ± 0.32 vs. 1.00 ± 0.00, *p* = 0.97). (G) WB bands of synaptic proteins. (H) Expression of PSD‐95 in the ipsilateral hippocampus reduced 7 days after TBI. However, the expression of vGlut1 remained unchanged (PSD‐95: 0.69 ± 0.23 vs. 1.36 ± 0.13, *p = 0.012; vGlut1: 3.40 ± 0.42 vs. 2.79 ± 0.92, *p* = 0.36).

Owing to vigorous phagocytosis 3 days postinjury, we assumed that the cognitive dysfunction observed 7 days after injury was related to the engulfment of synapses. To confirm this assumption, we performed Golgi staining of dendritic spines in the ipsilateral hippocampus. Spine density in both the CA1and CA3 regions was lower in the TBI group than in the sham group, especially in the CA1 region (Figure 3A–E). Because spines are the postsynaptic sites of most excitatory synapses, they can restrict postsynaptic density and limit signals.^21^, ^22^ We then performed WB and qRT–PCR to analyze the expression of excitatory synaptic proteins. Interestingly, 7 days after TBI, the transcription level of vGlut1 and PSD‐95 in the ipsilateral hippocampus did not change (Figure 3F), whereas the protein expression of PSD‐95 was reduced while that of vGlut1 remained unchanged. On the basis of this finding (Figure 3G,H), we suggest that TBI resulted in a reduction in hyperactive excitatory postsynapses, which may be attributed to protein degradation but not to a decrease in gene expression.

### 3.4 Significant phagocytosis in the ipsilateral hippocampus 7 days after TBI

**FIGURE 4:**
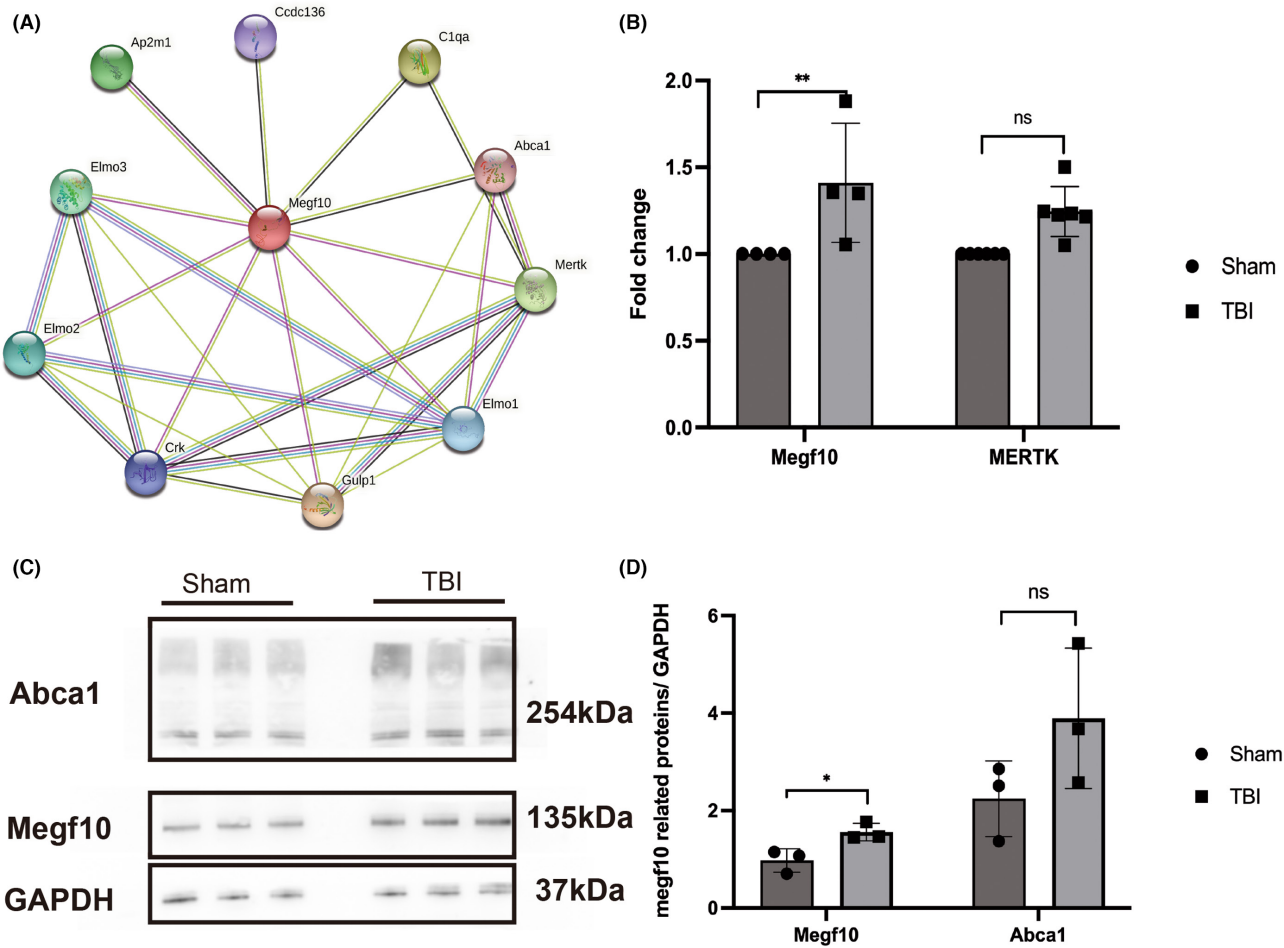
Transcription and protein levels of Megf10 were upregulated. (A) STRING illustrating Megf10 and relevant proteins, which were related to phagocytosis. (B) The transcription level of Megf10 was elevated 7 days after TBI (Megf10: 1.25 ± 0.14 vs. 1.00 ± 0.00, **p = 0.002; Mertk: 1.41 ± 0.34 vs. 1.00 ± 0.00, *p* = 0.54). (C) WB bands of Megf10 and relative proteins. (D) Expression of Megf10 was upregulated in the ipsilateral hippocampus 7 days post‐TBI (Megf10: 1.56 ± 0.18 vs. 0.97 ± 0.24, *p = 0.27; Abca1: 2.98 ± 1.97 vs. 1.05 ± 0.30, *p* = 0.17).

Numerous studies have reported active phagocytosis in the ipsilateral ischemic hemisphere, which is mediated by the Megf10 and Abca1 pathways.^23^ However, few studies have confirmed this finding following TBI. To verify the mechanism of phagocytosis, we used STRING to explore proteins correlated with the core protein of Megf10 (Figure 4A). Focusing on the main markers of Megf10, Abca1, and Mertk, we performed qRT–PCR and WB to examine key protein alterations. Expression of Megf10 was elevated in terms of both transcriptome and protein levels. Similarly, Abca1 and Mertk showed an increasing trend following TBI (Figure 4B–D). This indicated significant Megf10‐mediated phagocytosis in the ipsilateral hippocampus 7 days after TBI.

### 3.5 Megf10‐related engulfment of PSD‐95 by astrocytes after TBI

**FIGURE 5:**
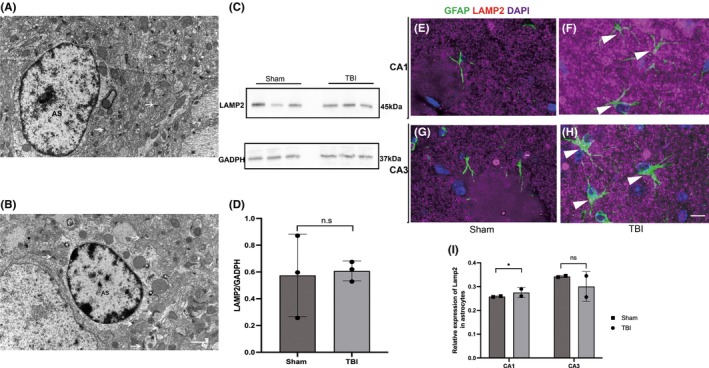
Astrocytes in the ipsilateral hippocampus showed activated phagocytic ability following TBI. (A and B) EM examination of the ipsilateral hippocampus in the sham and TBI groups, respectively. Scale bar: 1 μm. In the sham group (A), we detected astrocytes with normal morphology wrapped with synapses (white arrows). The heterochromatin in the nuclei was visible. However, in the TBI group (B), most astrocytes showed edema bodies, and proliferated lysosomes (white asterisks) were observed in the cytoplasm. In addition, astrocytes wrapped in additional synapses (white arrows) were observed. (C and D) WB results showed that total LAMP2 (a marker of lysosome) in the ipsilateral hippocampus was not elevated after TBI (0.57 ± 0.31 vs. 0.61 ± 0.74, *p* = 0.86, sham group vs. TBI group). (E and F) Co‐labeling of LAMP2 and GFAP in the CA1 region of the ipsilateral hippocampus in the sham and TBI groups, respectively. More co‐labeled LAMP2 and GFAP in the TBI group showed by the head of white arrows in (F). Scale bar: 5 μm. (G and H) Co‐labeling of LAMP2 and GFAP in the CA3 region of the ipsilateral hippocampus in the sham and TBI groups, respectively. Co‐labeled LAMP2 and GFAP in the TBI group were shown by the head of white arrows in (H). Scale bar: 5 μm. (I) Total LAMP2 did not increase following TBI; however, we observed an elevation in co‐labeled GFAP and LAMP2 in the CA1 region following TBI, which represented exuberant astrocytic phagocytosis (sham group vs. TBI group: 0.26 ± 0.00 vs. 0.35 ± 0.0, *p = 0.04 in CA1; 0.28 ± 0.21 vs. 0.31 ± 0.64, *p* = 0.63 in CA3).

**FIGURE 6:**
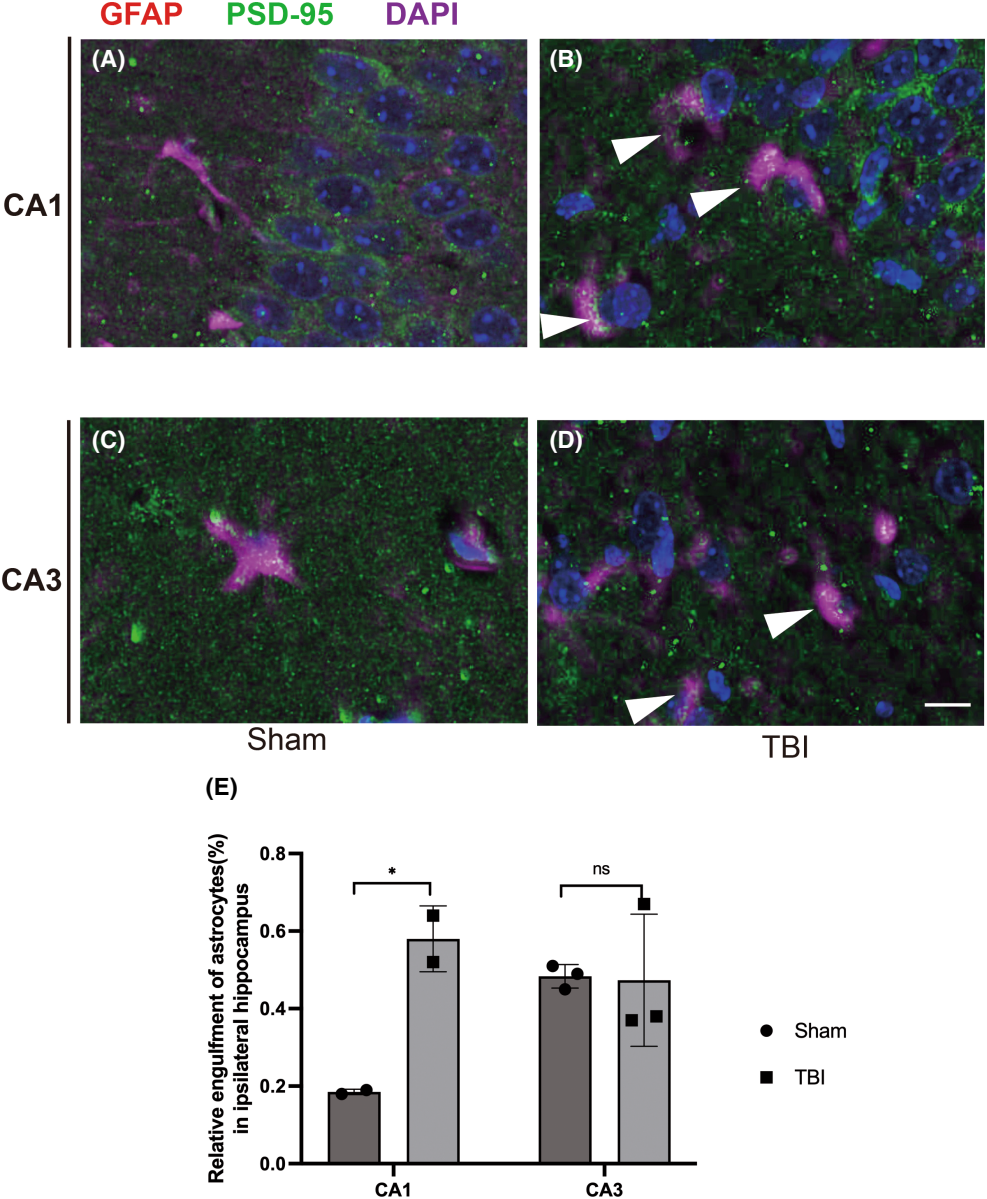
Astrocytes showed robust engulfment of PSD‐95 in the CA1 region of the ipsilateral hippocampus 7 days after TBI. (A and B) Co‐labeled astrocytes and PSD‐95 in the CA1 region in the sham and TBI groups, respectively. Co‐labeled PSD‐95 and GFAP in the CA1 of the TBI group were shown by the head of white arrows in (B). Scale bar: 5 μm. (C and D) Co‐labeled astrocytes and PSD‐95 in the CA3 region in the sham and TBI groups, respectively. Co‐labeled PSD‐95 and GFAP in the CA3 of the TBI group were shown by the head of white arrows in (D). Scale bar: 5 μm. (E) The engulfment ability of PSD‐95 in astrocytes was assessed by the co‐labeled ratio of GFAP to PSD‐95. We only detected a significant increase in the post‐TBI co‐labeled ratio in the CA1 region (0.58 ± 0.89 vs. 0.19 ± 0.01, *p = 0.03 in CA1; 0.47 ± 0.17 vs. 0.48 ± 0.03, *p* = 0.93 in CA3).

**FIGURE 7:**
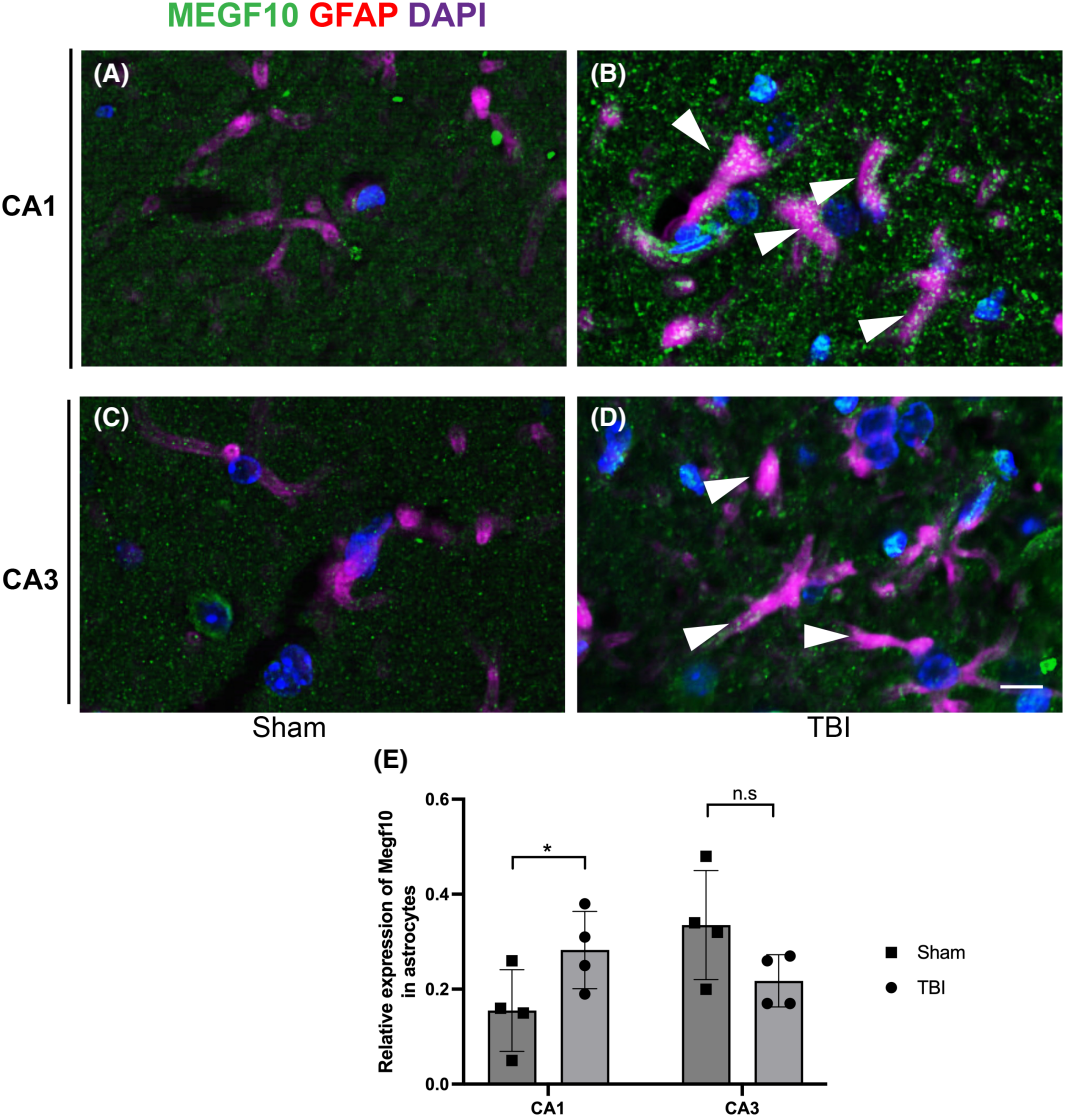
Megf10 was expressed in both astrocytes and neurons in the hippocampus, and there was increased expression of Megf10 in astrocytes in the CA1 region of the ipsilateral hippocampus following TBI. (A and B) Co‐labeling of astrocytes and Megf10 in the CA1 region in the sham and TBI groups, respectively. Elevated co‐labeled Megf10 and GFAP in the CA1 of the TBI group were shown by the head of white arrows in (B). Scale bar: 5 μm. (C and D) Co‐labeling of astrocytes and Megf10 in the CA3 region in the sham and TBI groups, respectively. Co‐labeled Megf10 and GFAP in the CA3 of the TBI group were shown by the head of white arrows in (D). Scale bar: 5 μm. (E) Co‐labeled Megf10 and GFAP showed an increased expression of Megf10 in astrocytes only in the CA1 region (0.34 ± 0.11 vs. 0.15 ± 0.85, *p = 0.04 in CA1; 0.22 ± 0.55 vs. 0.28 ± 0.81, *p* = 0.23 in CA3).

Previous studies have shown that astrocytic phagocytosis of synapses mediated by Megf10 in the adult hippocampus contributes to circuit homeostasis.^8^ Thus, we assumed that after TBI, Megf10‐related phagocytosis in astrocytes also significantly influences the stability of the hippocampus circuit. To confirm the phagocytosis of astrocytes, we conducted an EM experiment. Seven days after TBI, the proliferation of astrocytes was estimated in the ipsilateral hippocampus according to hypertrophic morphology and swelling bodies. Astrocytes were extensively infiltrated and wrapped around additional synapses. Moreover, an increase in lysosomes with second inclusion was observed in the astrocytes (Figure 5A,B). Although the total expression of LAMP2 did not increase significantly following TBI (Figure 5C,D), the elevated co‐location of GFAP and LAMP2 represented robust phagocytosis of astrocytes (Figure 5E–I). To visualize excitatory postsynaptic elimination by astrocytes, we co‐labeled GFAP and PSD‐95 with Alexafluor 594 and 488 in the ipsilateral hippocampus, and as expected, the co‐location of GFAP and PSD‐95 increased in the CA1 region of ipsilateral hippocampus in the post‐TBI group (Figure 6). This confirmed the elimination of postsynaptic materials by astrocytes. Simultaneously, the co‐labeled Megf10 and GFAP especially in the CA1 region showed an increase in the TBI group (Figure 7).

## 4 DISCUSSION

In the present study, we demonstrated a reduction in excitatory postsynaptic materials in the ipsilateral hippocampus during the subacute stage following severe TBI, which was consistent with poor performance on the cognitive tests. Our findings, alongside previous transcriptome data, suggested that exuberant exogenous phagocytosis is responsible for this process. Seven days post‐TBI, we identified phagocytic astrocytes containing secondary lysosomes that were wrapped around synapses. Furthermore, a significant increase in the co‐location of GFAP and PSD‐95 in the CA1 region suggested astrocytic engulfment of excitatory post‐synaptic proteins. We examined the phagocytic pathways and, unsurprisingly, both the transcription level and protein expression of Megf10 were elevated. Co‐immunofluorescence of GFAP and Megf10 demonstrated that the expression of Megf10 was spatially upregulated in astrocytes, exclusively in the CA1 region, which was related to astrocytic engulfment of PSD‐95. Therefore, we suggest that hyperactive astrogliosis and engulfment aggravate neural circuit destruction and cognitive impairment.

Cognitive sequelae after TBI result in long‐term deficits, and approximately 65% of patients with varying severity of TBI report cognitive impairments in memory, attention, and executive functions. In general, TBI severity and cognitive impairment have a linear relationship.^24^ The manifestation of TBI‐induced cognitive dysfunction varies widely, and the hippocampus is particularly prone to be affected. Normal hippocampal activity synchronizes activity across distal brain regions involved in cognitive processing, whereas hippocampal theta waves are decreased following lateral fluid percussion TBI.^25^ Numerous studies have demonstrated that impaired interconnections between hippocampus‐related pathways after TBI result in disruptions to cognitive processes. Moreover, the prefrontal cortex–hippocampus axonal tracts may be preferentially impaired following TBI and are likely to strongly influence work memory function. The consolidation of fear memory involves the axonal connections of the hippocampus and amygdala, the destruction of which causes anxiety problems following TBI.^26^ In our study, although the structure of the unilateral hippocampus remained intact, we detected a decrease in excitatory post‐synaptic proteins (PSD‐95), which was correlated with cognitive disorders.

To further investigate the mechanism underlying the reduction in excitatory post‐synaptic proteins in the ipsilateral hippocampus following TBI, we conducted transcriptome, qRT–PCR, and WB analyses. Interestingly, we found normal transcription but degradation in protein expression of excitatory post‐synaptic proteins. The transcriptome analysis showed that the phagocytic pathways re‐activated by the ipsilateral hippocampus involved with lysosomes played a pivotal role in the post‐TBI process. Previously, microglia were considered the only phagocytes in the CNS, and enhanced phagocytic capacity following brain injury was required to remove injured neurons, axons, and synapses during the early stage of injury. Additionally, numerous studies have demonstrated that the complement (C3 and C1q) and interleukin 13 mediate microglia engulfment activity following TBI.^27^, ^28^ However, recent studies have proposed that astrocytes also undergo phagocytosis. In the microglia ablation model, the re‐activation of astrocytes is a compensatory mechanism to maintain the stability of the CNS.^29^ Growing evidence has shown that astrocytes are enriched with engulfment‐related genes, such as TAM receptors, Megf10, and ABCA1.^30^ Furthermore, compared with microglial phagocytosis, astrocytic phagocytosis is initiated later but persists for longer, and astrocytes can engulf cellular debris, such as synapses, axons, and apoptotic cells.^31^ After brain injury, the cerebral environment undergoes dynamic structural and functional plasticity, and astrocytes proliferate to resistant apoptosis‐induced debris via the Fas‐ligand.^32^ Zhou et al.^30^ demonstrated that AXL‐mediated astrocytes phagocytose in response to active microglia following TBI. Moreover, during the subacute post‐stroke stage, astrogliosis actively engulfs synapses via the Megf10 and Mertk pathways, and the inhibition of astrocyte‐related engulfment of synapses improves neurobehavioral outcomes in patients with ischemic stroke.^7^ Similarly, we observed the proliferation of astrocytes with swollen bodies in the ipsilateral hippocampus, an increase in the astrocytic engulfment of excitatory postsynapses during the subacute stage post‐TBI, and an elevated expression of Megf10 in astrocytes.

Phagocytosis is a critical process involved in neural circuit homeostasis, in which apoptotic cells, synapses, and debris are dynamically removed. During the development of the CNS, phagocytosis is required for the refinement of synaptic connectivity, and the engulfment of redundant synapses contributes to memory and learning.^33^ Similarly, astrocytic pruning of synapses in the hippocampus in both the developing and adult hippocampus via Megf10 and Mertk helps to sustain a stable neural circuit.^10^ Throughout the lifespan, microglia constantly phagocytose apoptotic neural progenitor cells. However, following brain injury, phagocytosis of injured or apoptotic cells prevents the release of cytotoxic materials, and damaged myelin must be removed to enable axon regeneration. However, excessive or aberrant phagocytosis may result in disease or dysfunction. In the AD process, hyperactive complement‐mediated phagocytosis with microglia aggravates synaptic loss. Furthermore, extensive phagocytosis of live neurons during the post‐injury process results in a continuous pathological condition. Indeed, we observed hyperactive astrocytic phagocytosis of excitatory postsynaptic proteins in the ipsilateral hippocampus during the subacute stage following TBI and poor‐cognitive function.

Our study had several limitations. We associated the loss of synaptic proteins with poor neurobehavior; however, additional electrophysiological tests are needed to explore this further in the future. We found a positive relationship between astrocytic phagocytosis in the ipsilateral hippocampus and increased expression of Megf10 in astrocytes. We initially suggested that Megf10 contributes to the engulfment of excitatory postsynapses after TBI, especially in the CA1 region. However, further verification using Megf10‐knockout mice is needed to validate our results.

## 5 CONCLUSION

Megf10‐mediated engulfment of excitatory postsynaptic proteins by astrocytes in the CA1 region of the ipsilateral hippocampus aggravated cognitive dysfunction following severe TBI.

## CONFLICT OF INTEREST STATEMENT

The authors declare no competing interest.

## Supporting information


Figure S1.

## Data Availability

Data sharing not applicable – no new data generated, or the article describes entirely theoretical research
