# Supplementary figures and images for: Megf10‐related engulfment of excitatory postsynapses by astrocytes following severe brain injury
Source: CNS Neurosci Ther. 2023 Apr 20;29(10):2873–83. doi: 10.1111/cns.14223 (PMC10493650; doi:10.1111/cns.14223)

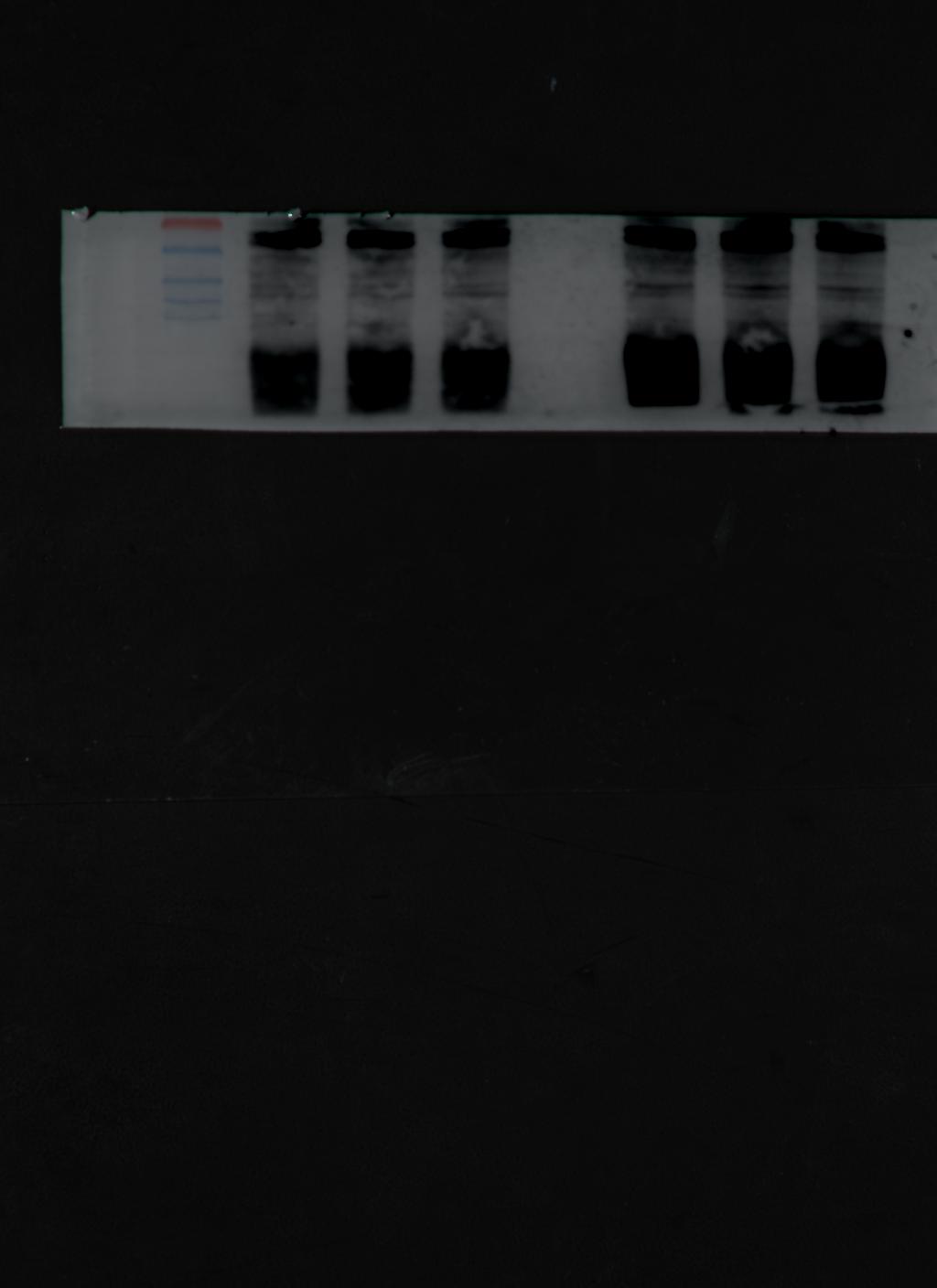

Supplement: Supplementary file 1 — Figure S1. [file CNS-29-2873-s001.zip › abca1.jpg]

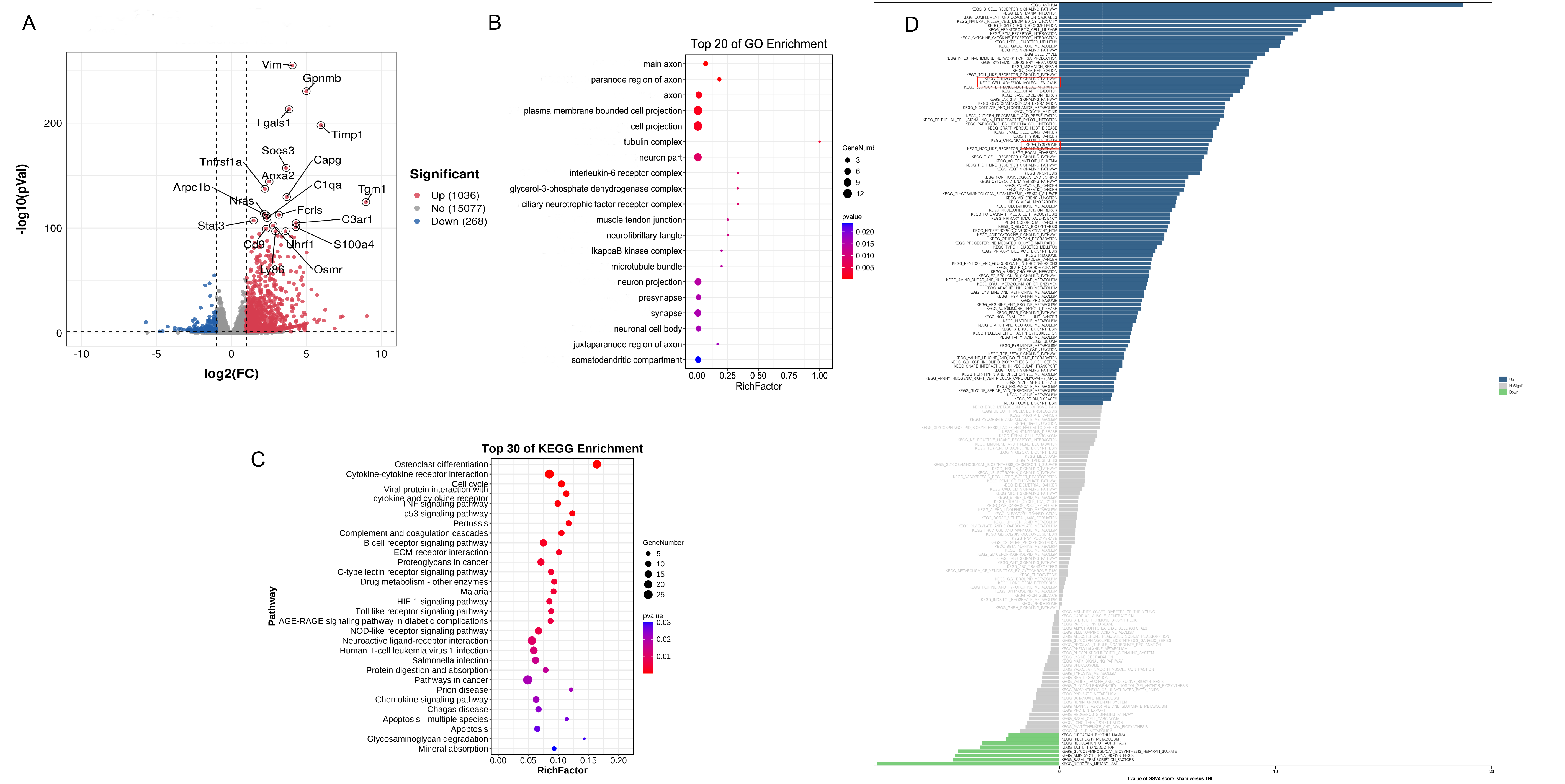

Supplement: Supplementary file 1 — Figure S1. [file CNS-29-2873-s001.zip › figure S1.tif]

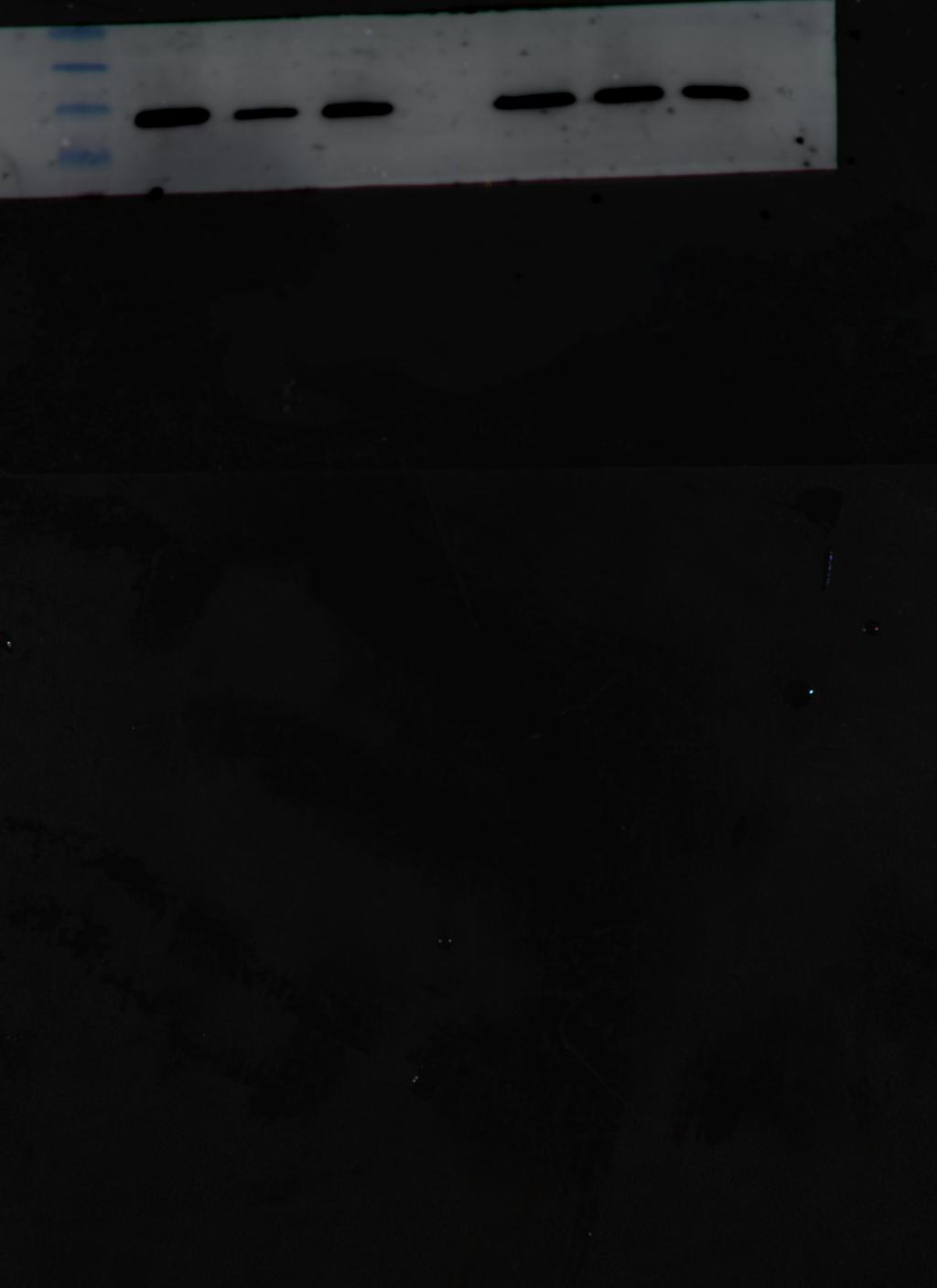

Supplement: Supplementary file 1 — Figure S1. [file CNS-29-2873-s001.zip › lamp2.jpg]

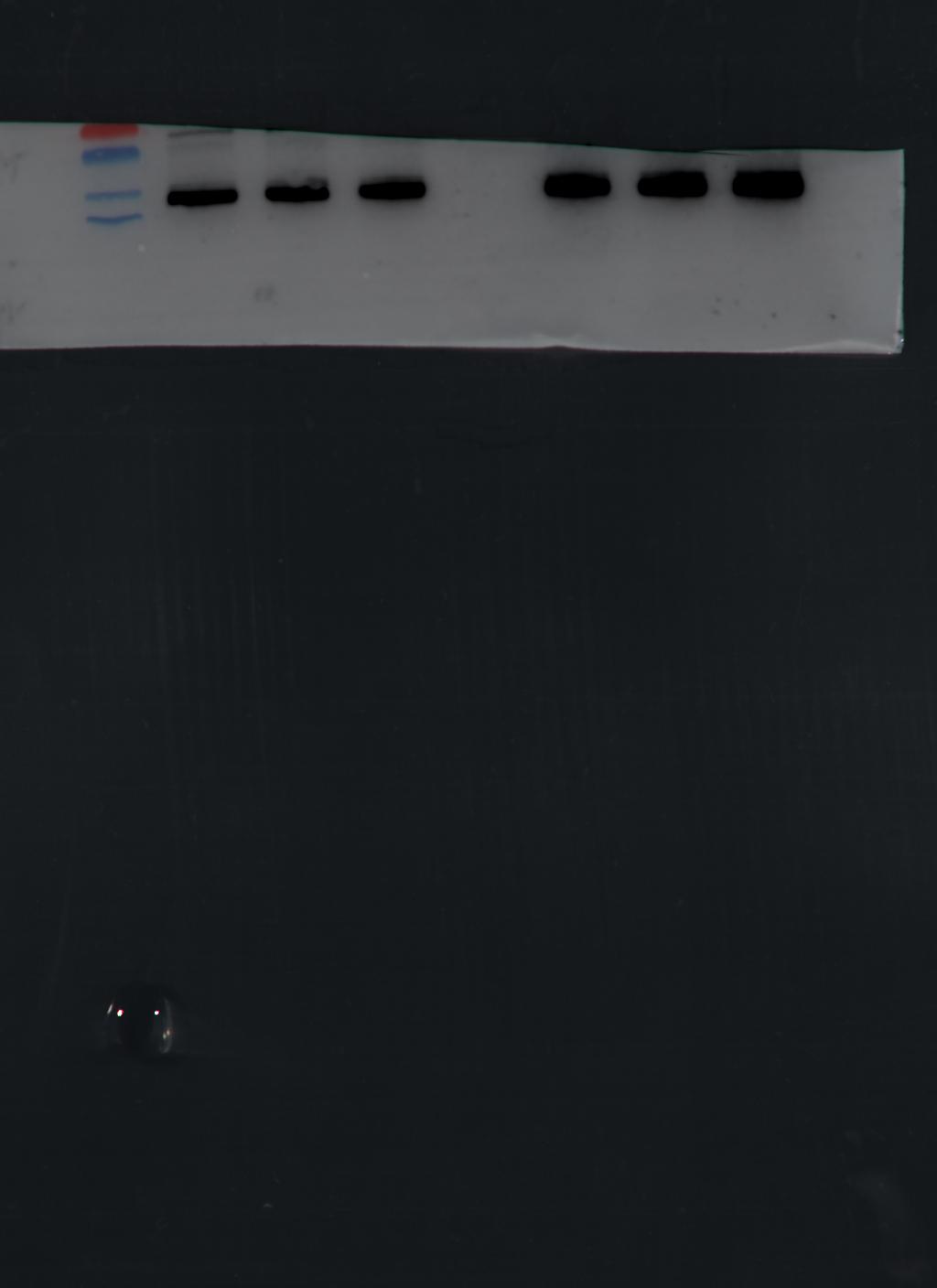

Supplement: Supplementary file 1 — Figure S1. [file CNS-29-2873-s001.zip › megf10.jpg]

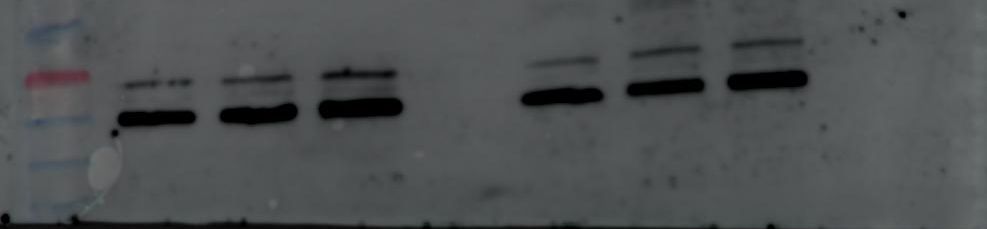

Supplement: Supplementary file 1 — Figure S1. [file CNS-29-2873-s001.zip › original-psd95 CNSNT-2022-943.R2.jpg]

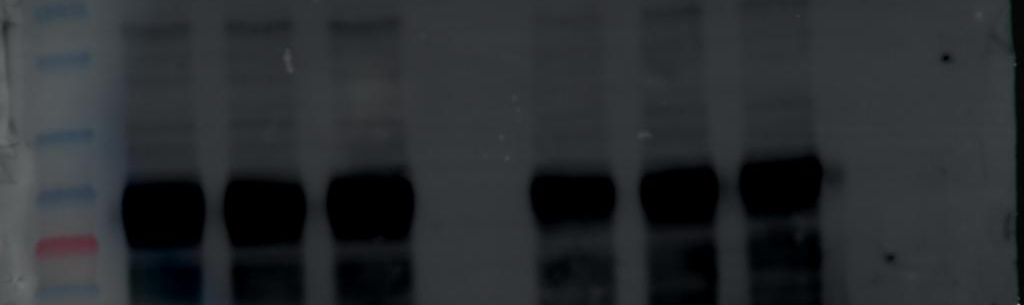

Supplement: Supplementary file 1 — Figure S1. [file CNS-29-2873-s001.zip › original-vglut1 CNSNT-2022-943.R2.jpg]
